# Potential Roles of Amiloride-Sensitive Sodium Channels in Cancer Development

**DOI:** 10.1155/2016/2190216

**Published:** 2016-06-15

**Authors:** Siguang Xu, Cui Liu, Yana Ma, Hong-Long Ji, Xiumin Li

**Affiliations:** ^1^Institute of Lung and Molecular Therapy, Xinxiang Medical University, Xinxiang, Henan 453003, China; ^2^Center for Cancer Research, Xinxiang Medical University, Xinxiang, Henan 453003, China; ^3^Department of Cellular and Molecular Biology, University of Texas Health Science Center at Tyler, Tyler, TX 75708, USA; ^4^Texas Lung Injury Institute, University of Texas Health Science Center at Tyler, Tyler, TX 75708, USA

## Abstract

The ENaC/degenerin ion channel superfamily includes the amiloride-sensitive epithelial sodium channel (ENaC) and acid sensitive ionic channel (ASIC). ENaC is a multimeric ion channel formed by heteromultimeric membrane glycoproteins, which participate in a multitude of biological processes by mediating the transport of sodium (Na^+^) across epithelial tissues such as the kidney, lungs, bladder, and gut. Aberrant ENaC functions contribute to several human disease states including pseudohypoaldosteronism, Liddle syndrome, cystic fibrosis, and salt-sensitive hypertension. Increasing evidence suggests that ion channels not only regulate ion homeostasis and electric signaling in excitable cells but also play important roles in cancer cell behaviors such as proliferation, apoptosis, invasion, and migration. Indeed, ENaCs/ASICs had been reported to be associated with cancer characteristics. Given their cell surface localization and pharmacology, pharmacological strategies to target ENaC/ASIC family members may be promising cancer therapeutics.

## 1. Introduction

Cancer is one of the leading causes of death worldwide. As reported by the World Health Organization, approximately 14 million new cancer cases were diagnosed in 2012, and 8.2 million deaths were cancer related. Of even greater concern, it is estimated that annual cancer cases will increase from 14 million in 2012 to 22 million within the next two decades. Although the traditional clinical treatments for cancer, including surgery, chemotherapy, and radiation therapy, have shown efficacy, side effects significantly reduce the quality of life for patients. Cancer relapse and treatment resistance further underscore the urgent need to identify novel targets for alternative therapies.

Ion channels are transmembrane proteins that have long been known to be involved in regulating a variety of physiological and pathological functions across biological membranes. Currently, approximately 13% of drugs used to treat various human diseases, including cardiovascular and neurological disorders, primarily target ion channels [1]. Indeed, the term channelopathies was coined to describe the ever growing number of diseases associated with ion channel function [2, 3]. Although cancer is still not cataloged as a channelopathy because of its complexity, it can be ascribed, at least in part, to ion channel malfunction [4]. An increasing number of studies showed that ENaC and ASIC channels are involved in various cancer cell behaviors, such as proliferation, apoptosis, invasion, and migration, and suggest that these channels are potential therapeutic targets for personalized cancer treatment [2, 4]. In this review, we summarize the latest findings on the role of ENaC and ASIC channels in cancer and discuss the mechanisms by which ENaC and ASIC channels may regulate cancer behaviors.

## 2. ENaC and ASIC in Proliferation and Apoptosis

Cell division and proliferation are key processes in cancer development and a role for ion channels in mediating cell proliferation has been reported in multiple types of cancer [4]. Indeed, earlier studies have shown that an influx of sodium ions (Na^+^) may stimulate a mitogenic signal and initiate the cell cycle and that inhibition of sodium transport can reduce the DNA synthesis required for cell proliferation [5, 6]. The role of the *α*-ENaC subunit in cell proliferation was first reported by Bondarava et al. [7], who showed that silencing *α*-ENaC reduced hypertonicity-induced Na^+^ currents by 60% and that HepG2 cell proliferation correspondingly decreased to approximately half that of the control. Moreover, transfection with *α*-ENaC siRNA caused a significant decrease in G1 phase cells and an increase in G2/M phase cells; the rate of apoptosis of HepG2 cells was also increased possibly because *α*-ENaC siRNA reduced Na^+^ currents, with the subsequent cell shrinkage (termed apoptotic volume decrease) being a hallmark of early apoptosis [8–10]. These results also revealed that *α*-ENaC plays a role in regulating Na^+^-induced cell proliferation, that its expression may be involved in tumor growth, and that it is a promising target for cancer treatment. Interestingly, although the sequence of *α*-ENaC has 35% homology at the amino acid level with *γ*-ENaC [11], Wang et al. [12] showed that inhibition of *γ*-ENaC is a possible mechanism for the antiapoptotic effects of hypotonic stress on IMCD cells, with p38, JNK, ERK, and the EGFR-JNK-PI3K pathway potentially stimulating sodium reabsorption in response to hypotonic stress via regulation of *γ*-ENaC [13–15]. Indeed, subcellular distribution of ENaC subunits varies: *α*-ENaC tends to be presented at the apical membrane of principal cells, whereas *β*-ENaC and *γ*-ENaC are dispersed throughout the entire cytoplasm. Whether these diverse distributions are involved in cancer development is unknown [16]. Although *δ*-ENaC shared 37% homology with *α*-ENaC and can form channels alone, or in combination with *β*-ENaC and *γ*-ENaC [17], Rooj et al. reported that knocking down *δ*-ENaC had no effect on cyclin-dependent kinase inhibitor expression in glioma [18]. In addition, serum- and glucocorticoid-regulated kinase 1, as a downstream effector of antiapoptotic phosphoinositide 3-kinase signaling, may interact with ENaC and specifically enhance ENaC activity [19]. In this process, glucocorticoid-induced leucine zipper 1 may help serum- and glucocorticoid-regulated kinase 1 to recruit ENaC by regulating the stability and localization of serum- and glucocorticoid-regulated kinase 1 [20]. Thus, understanding the role of ENaC family in proliferation and tumor growth requires further studies.

In addition to ENaCs, ASICs also belong to the epithelial/sodium channel degenerin (ENaC/DEG) superfamily and are predominantly distributed within the central and peripheral nervous systems [21]. Indeed, there is increasing evidence that ASICs are associated with malignant gliomas and that high-grade gliomas, such as glioblastoma multiforme, express multiple members of the ENaC/DEG family and characteristically display an amiloride-sensitive cation current, which is not seen in lower-grade gliomas or normal human astrocytes [22]. The malignant behavior, including high rates of proliferation, exhibited by glioma cells also requires large changes in cell volume, and volume recovery is important for a tumor cell [23, 24]. The spider toxin PcTx1, a selective ASIC1 blocker, inhibits ASIC1 but has no effect on other members of the ENaC/DEG family (tested mostly on homomeric channels) [18]. Both PcTX-1 and benzamil, an amiloride analog, induced cell cycle arrest of glioma D54-MG cells in G0/G1 phases, reduced cell accumulation in the S and G2/M phases, and upregulated the expression of the cell cycle inhibitors p21Cip1 and p27Kip1 [18]. Similar results were found with ASIC1 knockdowns, using U87MG and primary glioblastoma multiforme cells maintained in primary culture [18], and may be due to the effect of the ASIC1 knockdown on cell volume recovery via cation currents, because cells cannot divide in a shrunken state. Hu et al. further reported that blocking ASICs inhibited acid-induced apoptotic effect in chondrocytes in vitro, probably by inhibiting Ca^2+^ influx [25], as increased intracellular free Ca^2+^ is known to be involved in the development of apoptosis [26–29]. These results potentially explain the molecular mechanisms by which ASICs regulate the growth and apoptosis of cells.

## 3. ENaC and ASIC in Migration and Invasion

**Figure 1:**
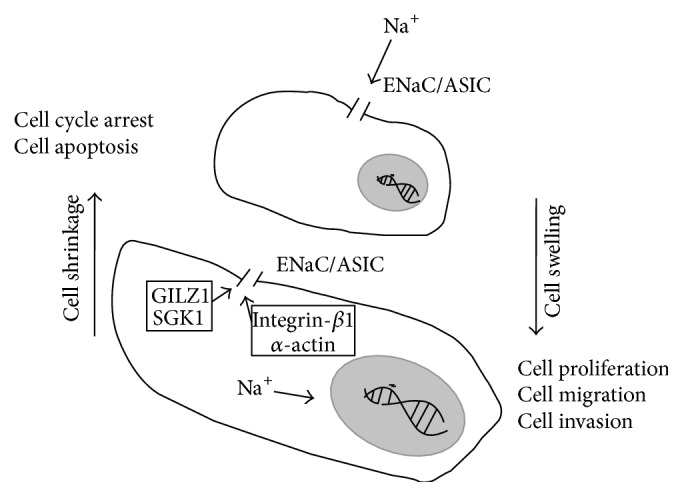
Contribution of ENaC/ASIC mediating Na^+^ current to cell volume in cancer cell development. ENaC/ASIC mediating Na^+^ influx promoted the proliferation, migration, and invasion of cancer cell, compared to blockage of this Na^+^ influx inducing cell cycle arrest and apoptosis of cancer cell.

Cancer cell migration and invasion are additional malignant behaviors in the progression of cancer. The ability of cancer cells to migrate allows them to metastasize in the body and invade other tissues or organs [30]. This process required the cell to become polarized and change its shape or stiffness, such as forming the lamellipodium and developing similar invadopodia, so that the cell can attach to the extracellular matrix and explore the surroundings via cell swelling, or retract the rear end of the cell via cell shrinking (Figure 1) [4]. These cell volume changes are normally regulated by ion transportation through various ion channels [31]. Malignant melanoma is the most aggressive neoplasm with severe metastatic potential and four homologous ENaC subunits (*α*-, *β*-, *γ*-, and *δ*-) were reported to be expressed in human malignant melanoma cells [32], but their relevance in malignancy and detailed function in malignant melanoma were unknown. Del Mónaco et al. further reported that they used antisense oligonucleotides directed against the *α*-ENaC subunit to decrease the migratory ability of BeWo cells [33], and Marino et al. further reported that aldosterone increased the ability of wound healing in BeWo cells in part through methylation of the ENaC [34]. Upregulation of *α*-ENaC can also stimulate trophoblast cell invasion ability by promoting the expression of matrix metalloproteinase 2, and knocking down *α*-ENaC expression reduces the invasion and migration abilities of HTR-8/SVneo cells [35, 36]. Additionally, induction of *β*-ENaC expression by heme oxygenase-1 promotes cytotrophoblast migration [37]. A recent study reported that *α*-ENaC and *γ*-ENaC were highly expressed in D54-MG human glioblastoma multiforme cells, compared with primary human astrocytes [38]. Additionally, knocking down *α*-ENaC or *γ*-ENaC affected the high P_K_^+^/P_Na_^+^ of D54-MG cells, and knocking down *α*-ENaC or *γ*-ENaC significantly inhibited D54-MG cell migration [38]. The inhibition of Na^+^ influx after channel knockdown and the subsequent inhibition of cell swelling required for lamellipodium expansion may be responsible for these observations. Furthermore, the established sodium gradient allows cells to take in important metabolic substrates and remove harmful metabolites and transportation of Na^+^ across the plasma membrane also enables cells to control their internal volume, including swelling or shrinkage [4]. Other studies have demonstrated that the subunits of *α*-ENaC and *γ*-ENaC regulated the cation current, including Na^+^, playing an essential biological function in glioma cell migration and invasion [23, 24, 39–44]. Tumor cells may use cation currents to recover volume, following migration through the brain parenchyma, and to move through the narrow extracellular spaces in the brain. Rooj et al. further reported physical and functional interactions between integrin-*β*1 and the amiloride-sensitive nonselective cation channel, composed of ASIC1, *α*-ENaC, and *γ*-ENaC, and further showed that knockdown of either integrin-*β*1 or *α*-actinin attenuated the amiloride-sensitive current [45], indicating that integrin-*β*1 and *α*-actinin may be involved in stabilizing the glioma cation channel complex and maintaining channel activity.

ASIC1 subunits also function similarly in promoting cancer cell migration. Sun et al. reported that CaMKII interacts with ASIC1 and colocalizes at the plasma membrane to form a functional complex that regulates glioma cell migration [46]. Here, the ASIC1 current plays an important role in glioma cell migration ability [18, 38], cell cycle progression [18], and volume regulation [47]. The Ca^2+^-sensitive kinase CaMKII may also play an important role in glioma biology by catalyzing the phosphorylation of ASIC1a at residues Ser478 and Ser479 and subsequently activating the ASIC channels [48]. Moreover, Ca^2+^ acts as a second messenger for supporting glioma cell migration [49], and ASIC1a may be permeable to calcium [50], suggesting that activated ASIC1 channels allow calcium to permeate and activate CaMKII, thereby regulating ASIC1 channels. In hepatocellular cancers, ASIC1*α* is also highly expressed and associated with advanced clinical stage. A moderately acidic extracellular environment can promote ASIC1*α* expression, and the migration and invasion of hepatocellular cells can be inhibited by silencing of ASIC1*α* expression [51], supporting evidence that tissue acidosis, an important feature of tumorigenesis, is associated with cancer invasion and angiogenesis [52]. In fact, many tumors showed a lower extracellular pH (pHe) environment compared with the corresponding normal tissue [53]. Rapid exposure of tumor cells to an acidic environment can transiently upregulate the proteolytic enzymes MMP-2 and MMP-9 and the proangiogenic factors IL-8 and VEGF-A [53, 54] and can also activate ASICs and induce Na^+^ influx, which subsequently affects cell volume regulation and membrane potential [55, 56], revealing that ASICs may be involved in regulating this process in cancer.

Nevertheless, not all ASIC family members play the same role in cancer. The amiloride-sensitive inward Na^+^ current, found in gliomas (also called glioblastoma multiforme or GBM), is absent in normal astrocytes and low-grade gliomas. Some studies show that GBM cells express this basal current due to the lack of ASIC2 in the plasma membrane [57]. Furthermore, although ASIC2a and ASIC3 are highly expressed in adenoid cystic carcinoma [58], surface expression of ASIC2 abolished the amiloride-sensitive inward Na^+^ current and promoted a reversion of a high-grade glioma cell to a more normal (i.e., nonmalignant) astrocytic phenotype [39]. Similar studies further reported that interaction between Hsc70 and ASIC2 can increase retention of ASIC2 in the endoplasmic reticulum in cells but that silencing Hsc70 may abolish the interaction, increase the surface expression of ASIC2 on cells, and inhibit cell migration, thereby also promoting reversion of a high-grade glioma cell to a more normal astrocytic phenotype [57, 59]. However, Liu et al. reported that knockdown of ASIC2a could increase the acidosis-induced cytotoxicity via the intracellular calcium overload in C6 glioma cells, revealing that ASIC2a could change the properties of ASICs, such as acid sensitivity and Ca^2+^ permeability, and subsequently affect the invasion and migration of the cells. Therefore, in contrast to ASIC1a, ASIC2a may play a protective role against the injury induced by extracellular acidosis in C6 cells [60]. Further investigation is needed to characterize more fully the role of each member of the ASIC family in cancer.

## 4. Prospective Studies

Studies on the role of other (ENaC/DEG) family members, such as HyNaC, DEL, PPK, UNC, and ACD [61], in cancer development have rarely been reported. However, increasing evidence suggests that ENaCs and ASICs of the ENaC/DEG family are associated with key aspects of cancer, and targeting members of this family is a potentially viable therapeutic approach. For example, recent study indicates that the acidic microenvironment (low extracellular pH, i.e., pHe-acidosis) can enhance the invasion activity of breast cancer cells in a ROS-AKT-NF-*κ*B-dependent manner. During this process, ASIC1 is required for acidosis-induced reactive oxidative species (ROS) production and NF-*κ*B activation, two key events for tumorigenesis. Moreover, regulation of ROS production by ASIC1 is specific to acidosis-induced cell invasion and tumor growth [62]. These data make ASIC1 an attractive target in breast cancer. On the other hand, most ENaC/ASIC proteins share a highly conserved structure: intracellular N- and C-termini and two membrane-spanning domains separated by a large extracellular domain [17]. Particularly, the pre-M2 region shows high homology between ENaC and ASIC. ENaC and ASIC both form amiloride-sensitive, non-voltage gated cation channels. ASIC channels tend to require higher doses of amiloride than ENaC. In addition, ASIC proteins (ASIC1, ASIC2, ASIC3, and ASIC4) can also form homo- and heteromultimeric channels that generally conduct Na^+^, although ASICs are activated by protons (H^+^) and ENaCs are highly selective for Na^+^ and Li^+^ [17]. All the common properties implicated that ENaC may play important roles in cancer development as ASIC did. Indeed, *γ*-ENaC was also reported to mediate ROS release in breast cancer [63], thus suggesting that targeting ENaC is also attractive in breast cancer. Tumor cells can use various mechanisms to remove intracellular acids to maintain physiological intracellular pH. These mechanisms include Na-driven proton extrusion, Na^+^/H^+^ exchangers. As a result, extracellular pH becomes more acidic. Acidity is harmful to normal cell, but tumor cells adapted well to acidic microenvironment during a long time of coevolution with the host [64]. ENaC/ASIC-mediated regulation of Na^+^ influx and reactive oxidative species (ROS) product may be involved in the sensing of external acidosis by tumor cells and downstream cascade. In addition, alterations of five ASIC genes were significantly related to poor patient survival [62]. To find out the mechanism of ASIC2, ASIC3, ASIC4, and ASIC5 on cancer development will be a specific question for next investigation. Particularly, ASIC5 is primarily expressed in the small intestine and the function of ASIC5 is not known [65]. Other significant questions also need to be addressed. Firstly, do factors in the tumor microenvironment, such as inflammation, immune cells, irradiation, chemotherapy treatment, or specific metabolins, induce activity of differential ENaC/DEG family members that can confer drug resistance? Secondly, are any mutations of ENaC/DEG family members involved in the oncogenic process and if so, are these mutated proteins potential targets for personalized medicine? Thirdly, can we use mutation of ENaC/DEG family members to establish various genetic models, for example, in the fruit fly, zebrafish, or mouse, to screen novel drug compounds that treat human cancer or other diseases? Fourthly, are there any drugs that when combined with ENaC/DEG family member inhibitors produce a synergistic effect in cancer therapy? Finally, are there any other novel proteins that can directly interact with and modify the function of ENaC/DEG family members during cancer development? Future research to address these and other questions will greatly enhance our understanding of the significance of the ENaC/DEG family in cancer and provide new avenues for the development of better therapies.
